# Protocol to study the antitumor and metabolic effects of intermittent fasting plus cisplatin and metformin in ovarian cancer PDX models

**DOI:** 10.1016/j.xpro.2026.104736

**Published:** 2026-07-27

**Authors:** Ludovica Perotti, Elena Capellini, Federica Guffanti, Anna Maria Ronchi, Daniela Corna, Domenico Cerullo, Cristina Matteo, Laura Sala, Eugenio Scanziani, Federica Maggi, Melissa Tiddia, Flavia Funari, Giovanna Damia, Laura Brunelli, Francesca Ricci

**Affiliations:** 1 Laboratory of Preclinical Gynecological Oncology, Department of Experimental Oncology, Istituto di Ricerche Farmacologiche Mario Negri IRCCS, Via M. Negri 2, 20156 Milan, Italy; 2 Laboratory of Clinical and Experimental Toxicology - Trace Elements Sector-Poison Control Center of Pavia - National Center for Toxicological Information- Scientific Clinical Institutes Maugeri SpA – SB, Via Salvatore Maugeri, 10, 27100 Pavia, Italy; 3 Unit of Experimental Models of Kidney Disease, Istituto di Ricerche Farmacologiche Mario Negri IRCCS, Centro Anna Maria Astori, Science and Technology Park Kilometro Rosso, Via Stezzano, 87, 24126 Bergamo, Italy; 4 Laboratory of Antitumor Pharmacology, Department of Experimental Oncology, Istituto di Ricerche Farmacologiche Mario Negri IRCCS, Via M. Negri 2, 20156 Milan, Italy; 5 Veterinary Medicine Department, University of Milan, Via Dell’Università 6, Lodi 26900, Italy, Mouse & Animal Pathology Lab (MAPLab), Fondazione UniMi, Viale Ortles, 22/4, Milan 20139, Italy; 6 Laboratory of Metabolites and Protein in Translational Research, Department of Environmental Health Sciences, Istituto di Ricerche Farmacologiche Mario Negri IRCCS, Via M. Negri 2, 20156 Milan, Italy

**Keywords:** Cancer, Metabolism, Model Organisms

## Abstract

Different dietary regimens are being explored as safe, low-cost approaches to enhance cancer therapy. This protocol describes the evaluation of antitumor and metabolic effects of intermittent fasting (IF) combined with cisplatin and metformin in an ovarian cancer patient-derived xenograft (PDX) model. Procedures include IF cycles, drug administration, antitumor activity assessment, drug quantification in plasma and tumors, toxicity evaluation, and immunohistochemical analyses.

For complete information on the generation and use of this protocol, please refer to Capellini et al.^1^

## Highlights

• Protocol to study intermittent fasting plus drug combinations in ovarian cancer PDXs
• Procedures for subcutaneous tumor implantation and *in vivo* antitumor evaluation
• Procedures for MET and DDP dosing and biochemical profiling in tumor and plasma samples
• Techniques for IHC staining on FFPE ovarian tumors

## Abstract (editor highlights)

Publisher’s note: Undertaking any experimental protocol requires adherence to local institutional guidelines for laboratory safety and ethics.

## Graphical abstract

**Figure:**
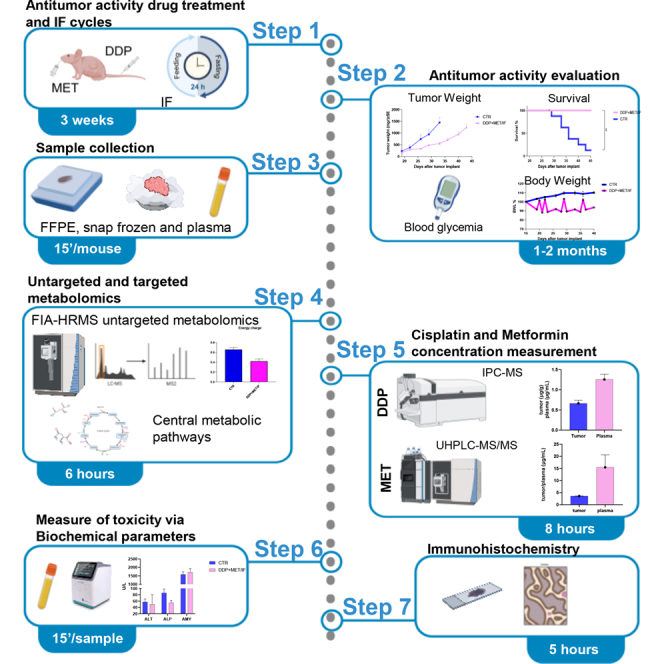

## Before you begin

This protocol describes the use of intermittent fasting in combination with a specific pharmacological therapy, including cisplatin and metformin, in an ovarian cancer (patient-derived xenograft) PDX model subcutaneously implanted in nude mice. Specifically, this protocol aims to: (1) evaluate the efficacy of adding a dietary regimen (i.e., intermittent fasting) to a pharmacological treatment (i.e., cisplatin and metformin); (2) investigate treatment-induced metabolic modulations at the tumor level under different experimental conditions; (3) assess the potential toxicity of the different treatment combinations; and (4) characterize the modulation of specific metabolic stress markers.

The reader will find detailed schedules and procedures for drug administration, either alone or in combination with the dietary regimen. The first part of the protocol focuses on experimental procedures involving animals, in particular tumor injection, antitumor activity evaluation, and sample preparation for downstream analyses. Then, a second part is related to more analytical studies, including the quantification of the drugs in the tumors and plasma following the different treatment schedules, the measurements of biochemical parameters in the plasma samples of mice as an indirect measure of drug toxicity (at kidney and liver levels), the evaluation of specific metabolites and energetic cofactors in tumors, and a protocol for the evaluation of the expression of specific proteins in formalin-fixed paraffin-embedded (FFPE) tumor samples by Immunohistochemistry (IHC).

Although the protocol was developed using the specific model and drugs employed in our recently published study,^1^ it can be adapted and adjusted for other tumor types and therapeutic agents.

### Innovation

In addition to standardized dietary regimens commonly applied in preclinical studies, this protocol incorporates the use of a specific dietary regimen in combination with drug treatment together with a comprehensive analytical evaluation. This includes the analysis of parameters related to cellular energetic status (key metabolites), measurement of drug levels within tumors and their distribution in plasma, assessment of biochemical parameters associated with treatment-related toxicity, and immunohistochemical evaluation of metabolic stress markers. The protocol provides step-by-step instructions, troubleshooting notes, and highlights critical steps.

### Institutional permissions

The IRFMN adheres to the principles set out in the following laws, regulations, and policies governing the care and use of laboratory animals: Italian Governing Law (D.lgs 26/2014; Authorisation n.19/2008-A issued March 6, 2008 by Ministry of Health); Mario Negri Institutional Regulations and Policies providing internal authorisation for persons conducting animal experiments (Quality Management System Certificate – UNI EN ISO 9001:2015 – Reg. N° 6121); the NIH Guide for the Care and Use of Laboratory Animals (2011 edition) and EU directives and guidelines (EEC Council Directive 2010/63/UE). National authorization by Ministry of Health for the presented protocol is 524/2020-PR. Prior to implementing this protocol, appropriate animal ethics approvals must be obtained for both the projects and all the personnel involved, according national requirements.

### Cisplatin and metformin preparation for *in vivo* antitumor activity

**Timing: 30 min**

1. Weight of drugs.
  a. Put on PPE (Personal Protective Equipment), specifically a double pair of gloves, glasses and a surgical mask.
  b. Use an analytical balance with a resolution of (at least) 0.001 g, and prepare a weighing paper to prevent the drug from adhering to the balance pan during weighing.
  c. Tare the balance and weigh the amount of drugs powder (cisplatin, DDP and metformin, MET) needed for the entire duration of the treatment in the experiment.

***Note:*** As both drugs reconstituted solutions are stable for the entire duration of the experiment is better to use a single drug solution to avoid weight bias during the experiment.

2. Dissolve the drug powders in their respective diluents. For DDP use 0.9% saline solution, and for MET use sterile water. Prepare DDP at a concentration of 0,4 mg/mL and MET at 40 mg/mL. Each mouse will receive 10μL of drug solution per gram of body weight.

### Preparation for inductively coupled plasma mass spectrometry analysis

**Timing: 2 h**

3. Check the release of metals from the test tubes (batch).
  a. Use 15 mL “*metal-free*” polypropylene tubes for samples and solution preparations.
  b. Check the release of metals from the walls of some test tubes (batch).
  c. Fill each polypropylene tube with approximately 10 mL of 10% (v/v) HNO_3_ prepared in ultrapure water and leave the solution in contact with the tube walls for 2 h at 20°C–25°C prior to ICP-MS analysis.
4. Reagent “blank analysis”.
  a. Use high purity reagents:
    i. Double-distilled water (resistivity ≥ 18,0 MΩ/cm at 25°C, conductivity ≤ 0,054 μS/cm, particulate matter < 0,1 μm).
    ii. Concentrated HNO_3_ (65%) *Suprapur.*
  b. Verify the degree of purity by ICP-MS analysis.

***Note:*** The analysis of metal release from plasticware and reagent blanks allows monitoring of potential contamination introduced during sample preparation and analysis. Blank signals were considered acceptable when below the method detection limit (MDL) or negligible compared to analyte concentrations in the samples. In the presence of detectable background contamination, blank subtraction was applied where appropriate.

5. Check the intrument’s “*Tuning”*
  a. Sensitivity: ^24^Mg (>6000 cps/1 μg/L), ^115^In (>80000 cps/1 μg/L), ^238^U (>60000 cps/1 μg/L).
  b. Stability: RSD% ^24^Mg, ^115^In, ^238^U.
  c. Oxides: intensity CeO/Ce < 3%.
  d. Double charges: intensity Ba++/Ba < 3%.
  e. Bkgd: 220.0 (<2 cps).

***Note:*** It is advisable to optimize the instrument by aspirating a tuning solution before analysis to verify that sensitivity, stability, oxide formation, and double-charge formation meet the method acceptance criteria. The tuning solution consisted of a commercially available ICP-MS tuning standard containing Be, Ce, Fe, In, Li, Mg, Pb, and U (1 μg/L each) prepared in 1% HNO_3_ using ultrapure water.

6. Tumor and plasma samples.
  a. Take tumor and plasma samples from the −80°C freezer and thaw them on ice (or at 4°C) for approximately 30–60 min until completely thawed to minimize metabolic alterations and potential analyte degradation.

***Note:*** It is necessary to carry out the conservation and subsequent treatment operations of the samples to guarantee their stability, homogeneity and the absence of contamination and loss of elements as much as possible.

### Preparation for ultra high-performance liquid chromatography coupled with tandem mass spectrometry analysis

**Timing: 2 h**

7. Preparation of Mobile Phases.
  a. Mobile Phase A (FM-A): 6 mM Ammonium Formate, pH 4.5 ± 0.02, with 0.1% Formic Acid (1 L).
    i. Weigh 378.36 mg of ammonium formate and transfer it into a clean volumetric flask.
    ii. Add approximately 800 mL of Milli-Q water and mix until the salt is completely dissolved.
    iii. Add 1 mL of formic acid to obtain a final concentration of 0.1% (v/v) concentration.
    iv. Adjust the solution to pH to 4.5 using small aliquots of ammonium hydroxide (NH_4_OH). The final pH must be 4.5 ± 0.02.
    v. Once the pH is stable, bring the solution to 1 L with Milli-Q water and sonicate for 10 min.
      ***Note:*** Prepare FM-A using LC–MS grade reagents and freshly prepared Milli-Q water to reduce background noise and ion suppression.
  b. Mobile Phase B (FM-B): 100 % Acetonitrile (CH_3_CN). Use LC–MS grade acetonitrile directly.
  c. Place the freshly prepared mobile phases on solvent tray, and activate the instrument’s purge/prime function for each solvent line for 5 min. Then, prime the autosampler needle wash (100% isopropanol) and seal wash (10% isopropanol solution) lines.
    ***Note:*** This step of proper purging ensures solvent lines are completely free of air bubbles and old mobile phase, reducing baseline noise, pressure fluctuations, retention time drift, and MS signal instability.
8. Column Installation.
  a. Install the analytical column Kinetex 2.6 μm XB-C18 100 A, 100 × 2.1 mm, along with a guard column (2.6 μm, 2.1 × 10 mm) of the same stationary phase (Phenomenex, Torrance, CA, USA).
  b. Set the column oven to 37°C and wait until thermal stabilization (20 min) before flowing mobile phase through.
  c. Start the column equilibration procedure with a low flow rate (0.1 mL/min) under the initial chromatographic conditions (1% FM-B) for 24 min (10 column volumes) to minimizes pressure shock.
  d. Increase gradually the flow rate to 0.4 mL/min. The normal backpressure expected should be approximately 400 bar, corresponding to about 5,800 psi.
9. System suitability test.
  a. Verify instrument performance using a system suitability sample prepared by dissolving 0.05 ng of MET, and 30 ng of ^2^H_6_-MET (as Internal Standard) in 350 μL of Acetonitrile.
  b. After injection of a solvent sample, the system suitability sample is injected in triplicate, and the following parameters are evaluated:
    i. Retention time repeatability: The percent coefficient of variation (CV%) of the absolute retention times across the three injections must be ≤ 15%.
    ii. Peak area repeatability: Calculate the %CV of the ratio between the peak area of the analyte and the peak area of the internal standard (IS) across the three injections. The %CV must be ≤ 15%.

***Note:*** The system suitability test (SST) may be conducted using similar parameters and acceptance criteria, that are similar and equally valid to those described and applied in the present method, such as resolution between critical peaks, signal-to-noise ratio, and theoretical plate count or peak symmetry (tailing factor).

10. Tumor and Plasma samples.
  a. Take the tumor and plasma study samples from the freezer and leave to thaw at 20°C–25°C.
  b. Thaw an adequate amount of control matrix (plasma and tumor homogenate) to prepare:
    i. blank sample (matrix without analyte and without IS).
    ii. zero sample (matrix with internal standard only).
    iii. Six calibration standards (6-point calibration curve) prepared by spiking control matrix.
    iv. At least two replicates for each of the three QC levels (low, medium, high).
  c. Weight tumor tissues and transfer them into homogenization tubes.
  d. Add cold phosphate-buffered saline (PBS) at a ratio of 1 g tissue to 3 mL buffer.
  e. Homogenize the samples using a Turrax homogenizer.

***Note:*** Control plasma is prepared by collecting mouse blood into heparinized tubes, followed by centrifugation at 4,000 rpm for 10 min at 4 °C. The plasma supernatant is collected and stored at −20 °C until analysis. The control tumor homogenate is prepared from tumors excised from untreated mice.

### Immunohistochemistry reagent preparation

**Timing: 30 min**

11. Deparaffinization and Heat Induced Epitope Retrieval (HIER).
  a. Prepare heat-resistant containers (e.g., BioSB: Plastic Staining Dish, BSB7009) filled with Buffer H, pH 9.
  b. Prepare an equal number of additional containers (e.g., BioSB: Plastic Staining Dish, BSB7009) filled with distilled water (dH_2_O).
  c. Place all containers in a water bath with a support (e.g., BioSB: Staining Dish Support, BSB7086; TintoRetriever Digital Pressure Cooker, BS 7008) filled with dH_2_O, which will be used for water-bath heating during the antigen retrieval procedure.
12. Blocking of endogenous peroxidase activity.
  a. Prepare a solution of 3% hydrogen peroxide (H_2_O_2_) in distilled water (dH_2_O) to block endogenous peroxidase activity, as a peroxidase-based chromogen (DAB) will be used for signal detection.
13. Blocking of non-specific binding sites.
  a. Prepare a solution of 10% normal animal serum (selected according to the host species of the secondary antibody) diluted in PBS 1× to reduce non-specific antibody binding.
  b. The same 10% normal serum is also used as the diluent for primary antibodies.
14. Other reagents.
  a. Each primary antibody is diluted (see key resources table) in the same 10% normal serum solution used for the blocking of non-specific binding sites.
  b. Biotinylated secondary antibody is diluted 1:200 in PBS 1×.
  c. Avidin–Biotin Complex (ABC) solution is diluted 1:150 in PBS 1×.
  d. DAB (3,3′-diaminobenzidine) is prepared at a concentration of 1 drop per 1 mL of diluent.
  e. PBS 1× containing 0,025% of Tween20 to be used for all washing steps.
15. Nuclear counterstaining.
  a. Counterstain sections with Mayer’s Hematoxylin.
16. Dehydration and mounting.
  a. To perform dehydration of the slides after IHC, the following reagents are required: 95% ethanol, 100% ethanol, and 100% xylene.
  b. Mount coverslips using Micromount (Diapath, 060200) or an equivalent mounting medium.

## Key resources table

**Table:** 

REAGENT or RESOURCE	SOURCE	IDENTIFIER
**Antibodies**		

Glutathione Peroxidase 4-GPX4 (1:5000)	Abcam (Rabbit monoclonal)	ab125066; [EPNCIR144]; lot#1000287-25
4-hydroxynonenal-4-HNE (1:4000)	Bioss Antibodies (Rabbit monoclonal)	bs-6313R; lot#BC07253787
Malondialdehyde-MDA (1:1500)	LS Biosciences (Rabbit polyclonal)	LS-C72018; lot#236315
anti-LAMP2 (1:20000)	Abcam (Rabbit monoclonal)	ab199946; [EPR1952]; lot#1027879-26
p62 (1:100)	Enzo Life Sciencies (Rabbit polyclonal)	BML-PW9860-0025
ACSL5 (1:100)	Abcam (Rabbit polyclonal)	ab272556; lot#1003177-6
Ki67 sp6 (1:500)	Thermoscientific (Rabbit monoclonal)	#RM-9106-S
Cleaved Caspase 3 9661 (1:2000)	Cell Signaling (Rabbit polyclonal)	Asp175, [D175]; #9661S; lot#47
Goat anti-rabbit IgG (1:200)	Vector Laboratories, USA	BA-1000

**Chemicals, peptides, and recombinant proteins**		

Cis-Diammineplatinum(II) dichloride DDP	Sigma-Aldrich	P4394
Metformin	Sigma-Aldrich	D150959
Methanol RS - For LC/MS	Carlo Erba reagents	414832
Acetonitrile RS - For LC/MS	Carlo Erba reagents	412342
Water RS - For LC/MS	Carlo Erba reagents	412112
Cytidine 5′-monophosphate	Sigma-Aldrich	C1131-500mg
Guanosine 5′-monophosphate disodium salt hydrate	Sigma-Aldrich	G83377-500MG
Uracil	Sigma-Aldrich	U0750-5g
Adenosine-5′-monophosphate AMP	Carlo Erba	A102790050-5g
Hypoxanthine	Sigma-Aldrich	H9377-1g
Uridine	Sigma-Aldrich	U3750-1g
Thymine	Sigma-Aldrich	T0376-5g
Inosine	Sigma-Aldrich	I4125-1g
Guanosine	Sigma-Aldrich	75903-100g
Xanthosine dihydrate	Sigma-Aldrich	X0750-5g
Thymidine	Sigma-Aldrich	89270-1g
Adenine	Sigma-Aldrich	A8626-1g
Cytidine	Sigma-Aldrich	C4654-1g
Adenosine	Sigma-Aldrich	A9251-1g
2′-Deoxycytidine	Sigma-Aldrich	D3897-100mg
Uric Acid	Sigma-Aldrich	U0881-10g
Xanthine	Sigma-Aldrich	X0626-5g
Guanine	Sigma-Aldrich	G11950-10g
Niacinamide	Sigma-Aldrich	72340-100g
D-(+)-Glucosamine hydrochloride	Sigma-Aldrich	G4875-10mg
D-Gluconic acid sodium salt	Sigma-Aldrich	G9005-500g
L-Threonic acid hemicalcium salt	Sigma-Aldrich	380644- 5g
Sucrose	Sigma-Aldrich	84097-250g
Nicotinic Acid	Sigma-Aldrich	72309-100g
Choline chloride	Sigma-Aldrich	C7017 – 10mg
D-Pantothenic Acid hemicalcium salt	Sigma-Aldrich	21210- 5g
Pyridoxal hydrochloride	Sigma-Aldrich	P9130-500mg
4-Aminobenzoic Acid	Sigma-Aldrich	01973-100mg
Riboflavin	Sigma-Aldrich	47861- 1000 mg
Biotin	Sigma-Aldrich	B4501-100mg
Ergocalciferol	Sigma-Aldrich	95220-1g
L- Ascorbic Acid	Sigma-Aldrich	A5960-10mg
Cyanocobalamine	Sigma-Aldrich	V2876-100mg
Pyridoxine	Sigma-Aldrich	P5669-5g
(±)-α-Lipoic Acid	Sigma-Aldrich	62320-5g
DL-α-Tocopherol acetate	Sigma-Aldrich	T3376-5g
Cystine	Sigma-Aldrich	C4654-1g
L-Asparagine	Sigma-Aldrich	A0884-10mg
L-Aspartic Acid	Sigma-Aldrich	A9256-100g
L-Serine	Sigma-Aldrich	S4500-1g
L-4-Hydroxyproline methyl ester hydrochloride	Sigma-Aldrich	30681- 5g
Glycine	Sigma-Aldrich	G7126-10mg
D-Glutamine	Sigma-Aldrich	G9003-1g
L-Cysteine	Sigma-Aldrich	C7352-10 mg
L-Threonine	Sigma-Aldrich	T8625-1g
L-Methionine Sulfoxide	Sigma-Aldrich	M1126-1g
L-Glutamic Acid	Sigma-Aldrich	G1251-100 g
D-Alanine	Sigma-Aldrich	A7377 -5g
L-Citrulline	Sigma-Aldrich	C7629-1g
L-Ornithine monohydrochloride	Sigma-Aldrich	O2375-10mg
L-Proline	Sigma-Aldrich	P0380-10mg
D-Lysine	Sigma-Aldrich	L8021-1g
L-Histidine monohydrochloride monohydrate	Sigma-Aldrich	H8125- 5g
L-Arginine monohydrochloride	Sigma-Aldrich	A5131-10g
N-Acetyl-L-aspartic acid	Sigma-Aldrich	00920- 5g
4-Aminobutyric Acid	Sigma-Aldrich	PHR3865-100mg
L- Glutathione reduced	Sigma-Aldrich	G4251- 10 mg
L-Valine	Sigma-Aldrich	V0500-1g
D-Methionine	Sigma-Aldrich	M9375- 5g
N-Acetylcysteine amide	Sigma-Aldrich	A0737- 5g
L-Tyrosine	Sigma-Aldrich	T3754- 50g
L-Isoleucine	Sigma-Aldrich	I2752-1g
D-Leucine	Sigma-Aldrich	855448-2,5g
D-Phenylalanine	Sigma-Aldrich	P1751-5g
L-Kynurenine	Sigma-Aldrich	K8625-25mg
DL-Tryptophan	Sigma-Aldrich	T3300-5g
Cystathione	MedChemExpress	56-88-2 – 1mg
L-2-Aminoadipic acid	Sigma-Aldrich	55398
5-Oxo-DL-proline-d5	LGC group	TRC-O858972-1MG
Hexose (D-(+)-Glucose)	Sigma-Aldrich	G8270-250mg
Malate	Sigma-Aldrich	06994 -100ml
Sodium Pyruvate	Sigma-Aldrich	P2256- 10mg
Sodium L-Lactate	Sigma-Aldrich	L70022-5g
Succinic acid	Sigma-Aldrich	S3674- 100g
Fumaric acid	Sigma-Aldrich	798673 – 100g
Glyceric Acid (D-Glyceric acid calcium salt dihydrate)	Sigma-Aldrich	367494- 1g
2-(2-Aminoethoxy)ethanol	Thermo Fisher	L18897.22
Ethylenediamine	Sigma-Aldrich	E26266-5ml
Putrescine	Sigma-Aldrich	51799-100mg
O-Phosphoethanolamine	Sigma-Aldrich	P0503- 10mg
Histamine	Sigma-Aldrich	H7125- 1g
Flavin Adenine Dinucleotide disodium salt hydrate (FAD)	Sigma-Aldrich	F6625- 10mg
Riboflavin 5′monophosphate sodium salt hydrate (FMN)	Sigma-Aldrich	F2253- 10 mg
β- Nicotinamide Mononucleotide	Sigma-Aldrich	N3501-25mg
Guanosine 5′-diphosphate sodium salt (GDP)	Sigma-Aldrich	G7127-10mg
Guanosine 5′-triphosphate sodium salt hydrate (GTP)	Sigma-Aldrich	10106399001- 250mg
Adenosine 5′-diphosphate sodium salt (ADP)	Sigma-Aldrich	A2754-100mg
Adenosine 5′-triphosphate disodium salt solution (ATP)	Sigma-Aldrich	A6559-25 μmol
β-Nicotinamide adenine dinucleotide hydrate (NAD)	Sigma-Aldrich	N1511-250mg
β-Nicotinamide adenine dinucleotide, reduced disodium salt (NADH)	Sigma-Aldrich	N8129-5mg
β-Nicotinamide adenine dinucleotide phosphate disodium salt (NADP)	Sigma-Aldrich	NADP-RO-100mg
Acetic acid solution	Sigma-Aldrich	45754
Ammonium formate	Sigma-Aldrich	156264
Nitric Acid, HNO_3_ 65% *Suprapur*®	Avantor-Merck Chemicals	1.00441.0250
Platinum Standard Solution_ICP-MS standard, 10 mg/L Pt in 2% HCl	Avantor-VWR Chemicals	85589.180
NexIon Setup Solution, *TruQ™* ms	PerkinElmer Standards	PE #: N8145051
NexIon Dual Detector Calibration Solution, *TruQ™* ms	PerkinElmer Standards	PE #: N8145059
NexIon KED Mode Setup Solution, *TruQ™* ms	PerkinElmer Standards	PE #: N8145052
^2^H_6_-metformin	Alsachim SAS, Illkirch, Francia	Cat # C1160 Batch # DH-ALS-14-013-P5
Acetonitrile	CARLO ERBA Reagents S.r.l., Milan, Italy	Cat # 412371000 Batch # P3L715253L
Ammonium formate	Sigma-Aldrich, Merck Spa, Milan, Italy	Cat # 70221-25G-F Batch # BCCM8503
Dewax and HIER Buffer H	Epredia	TA-999-DHBH
A-PAP PEN	Liquid Daido Sangyo Co.,Ltd	11-100; lot#24018
Hydrogen peroxide 30%	Sigma Aldrich	1072981000; lot#K54213798-230
VECTASTAIN® Elite ABC-Peroxidase Kit Standard	Vector Laboratories, USA	PK-6100
ImmPACT® DAB, Peroxidase DAB Substrate Kit	Vector Laboratories, USA	SK-4105
Mayer Hematoxylin	Diapath	C0303
Eosin G or Y polychromatic 1% aqueous	Diapath	C0363

**Critical commercial assays**		

Contour XT	SCENSIA Diabetes Care	#84190994

**Experimental models: Organisms/strains**		

Mouse: Female athymic nude Foxn1nu 5–6 weeks old	Envigo RMS srl	N/A

**Software and algorithms**		

EASY-FIA	Morabito et al^2^	GitHub at https://github.com/AMrbt20/EASY-FIA/
GraphPad Prism (v. 9.2)	Dotmatics	
MetaboAnalyst 6.0	www.metaboanalyst.ca	
mixOmics package (version 4.3.242)	https://mixomics.org/	
*Syngistix™* Software for ICP-MS V3.4	PerkinElmer Scientific	https://www.perkinelmer.com
*Syngistix™* Software for ICP-MS V3.5 HF1	PerkinElmer Scientific	https://www.perkinelmer.com

**Other**		

Discovery HS F5-3 (2.1 mm I.D. × 150 mm, 3 μm) column	Sigma-Aldrich	567503-U
BD Vacutainer® Citrate 2.7ml 13 × 75mm	Becton Dickinson	363048
X-Bridge BEH Amide (2.1 mm I.D. × 150 mm, 3.5 μm) column	Waters	186004861
DRC-ICP-MS *NexIon* 2000B PerkinElmer	PerkinElmer Instruments	https://www.perkinelmer.com
Microwave system CEM, model *MARS-Xpress*	CEM (Corp, Matthews, NC, USA)	https://www.cem.com
*Milli-Q™* deionizing system - High purity water	Millipore (Bedford, MA USA)	https://www.merckmillipore.com
UHPLC-MS/MS system: ExionLC™ SCIEX Triple Quad 3500	AB SCIEX Italia s.r.l., Milan, Italy	5062231
Kinetex 2.6 μm XB-C18 100 Å column (100 × 2.1 mm)	Phenomenex, Torrance, CA, USA	Part No.:00D-4496-AN 00D-4497-AN
SecurityGuard ULTRAKinetex security guard ULTRA Cartridges, UHPLC C18 2.1mm	Phenomenex, Torrance, CA, USA	Part No.: AJ0-8782
Element RC Rotor Comprehensive Plus	Scil Animal Care (Italy)	#108668
Autostainer	Epredia	480S-A8050007
Instantpot/ TintoRetriever Digital Pressure CookerCooker (or equivalent)	BioSB	BSB7008
Staining Dish Support (or equivalent)	BioSB	BSB 7086
Plastic Staining Dish (or equivalent)	BioSB	BSB 7009
Induction hot plate	\	\

## Step-by-step method details

### Tumor harvesting and implantation

**Timing: 30 min**

This section describes how to perform tumor implantation for *in vivo* antitumor activity studies. In this study we use stabilized models that were previously obtained and stabilized as per Ricci et al, 2014^2^and Ricci et al, 2019.^3^

1. Preparation for tumor fragments implants in nude mice.
  a. Euthanize the donor animal by exposure to increasing concentrations of CO_2_ (or other approved protocol).
  b. Disinfect the tumor area.

***Note:*** In our study, we used female nude mice, as the experimental model involves a female gynecologic patient-derived xenograft (PDX) model.

**CRITICAL:** Allow mice to acclimatize for one week after arrival at the facility before starting any experiments. Environmental and experimental distress can alter tumor engraftment and drug toxicity/response of mice. Minimize stress-related effects due to environmental causes (i.e. noise, vibrations, and changes in ambient temperature), and by using environmental enrichment in cages.

All experiments conducted on PDX models require the use of immunodeficient mouse strains. For this reason, all procedures described below that involve the handling of nude mice must be carried out under a sterile hood, using sterile and previously sanitized instruments.

2. Tumor excision.
  a. Make a small incision on the skin close to the tumor.
  b. Carefully detach the skin from the tumor.
  c. Excise the tumor and place it in a Petri dish containing saline solution to maintain tissue hydration.
3. Tumor processing.
  a. Remove the external capsule of the tumor.
  b. Discard necrotic or poorly cellularized portions.
  c. Mince the tumor into homogeneous fragments (n= number of mice to be injected) of approximately 3 mm in diameter.
4. Fragment preparation.
  a. Using forceps, place each tumor fragment inside a trocar.
5. Recipient animal preparation.
  a. Anesthetize recipient animals with isoflurane (3% for induction; 1%–2% for maintenance).
  b. Disinfect the target implantation area.
6. Tumor implantation.
  a. Make a small incision (a few millimeters) on the skin of animal’s flank using scissors.
  b. Insert the trocar subcutaneously to the desired implantation site (flank).
  c. Ensure the fragment is positioned properly to avoid extrusion during trocar removal.
7. Post-procedure care.
  a. Return the animal to its cage.
  b. Monitor the animal until full recovery from anesthesia.

Repeat from step 4 to step 7 for each mouse to be injected with the tumor fragment.

***Note:*** For the number of mice to be injected, consider tumor engraftment percentage, number of mice needed for statical significant analysis of antitumor activity and survival evaluation, and the number of mice for downstream analysis.

### Antitumor activity

**Timing: 1–2 months**

**Timing: 24 h per cycle**

This section outlines the procedures for evaluating antitumor activity of a subcutaneous ovarian cancer PDX model.

These steps describe how to randomize animals into the different treatment groups and how to perform drug administration as well as the addition of intermittent fasting (IF). DDP is given intravenously, while MET *per os*.

8. Randomize animals according to tumor volume and body weight to ensure equal distribution of tumors and mice across each treatment group (8 mice).

**Figure 1:**
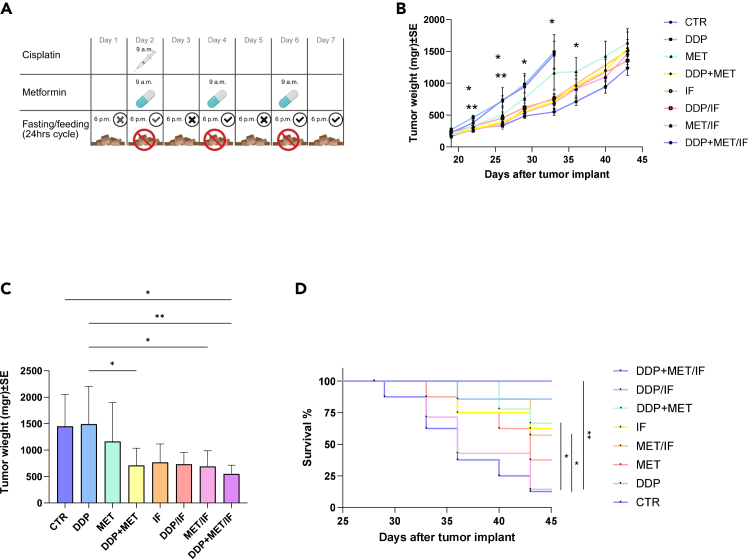
Antitumor activity representative results (A) Schedule treatment. (B) Antitumor activity of the combination of IF associated, or not, with single or combination of drugs. The mean±SE of tumor weight for each group of treatment (8 mice per group) is represented. Graph starts from randomization time. Red arrows indicate the starting of weekly treatment. Day 22: ∗ is DDP vs IF; ∗∗ are DDP vs MET/IF; Day 26: ∗ is DDP vs IF; ∗∗ are DDP vs DDP+MET/IF; Day 29: ∗ is DDP vs DDP+MET/IF; Day 33: ∗ is DDP vs DDP+MET/IF; Day 36: ∗ is DDP vs DDP+MET/IF (Turkey test). (C) The mean of the tumor weight±SE of each group at the time of best response (day 33) is reported. ∗ p < 0,05; ∗∗ p < 0,005 (Tukey test). (D) Kaplan Meier curves for survival % of the different groups of treatments up to day 45 from tumor implant. ∗ p < 0,05; ∗∗ p < 0,005 (Mantel-Cox test). Figure reprinted with the permission from Capellini et al, 2026.^1^

***Note:*** Each combination evaluated requires a corresponding control group. In our study, we included a control/vehicle-treated group, a DDP-only group, a MET-only group, a DDP+MET combination group, and the same groups with the addition of an IF regimen (Figure 1A).

9. Intravenous administration of DDP.
  a. Syringe preparation: draw the exact volume of solution to be injected into the syringe, taking care to remove any air bubbles, as they may alter the administered volume as well as be fatal when injected intravenously in mice.
  b. Remove the animal from its cage and place it under an infrared lamp for a few seconds, exposing the tail to heat while protecting the rest of the body.
  c. Immobilize the animal using a restrain system.
  d. Disinfect the tail with a cotton swab soaked in disinfectant and apply gentle traction to immobilize it.
  e. Identify one of the lateral tail veins and insert a 26G, 1 ml syringe needle 4–5 mm into the vein, keeping the needle parallel to the vein.
  f. Slowly inject the drug.
  g. Withdraw the needle and apply gentle pressure to the injection site with cotton to promote hemostasis.
  h. Return the animal to its cage.

***Note:*** In our experiment we use a dose of DDP of 4 mg/kg (0,4 mg/mL, administered 10μL per gram of mouse body weight), every 7 days for 4 weeks.

10. Oral Administration (*per os*) of MET.
  a. Syringe preparation: Attach a gavage tube to a 1 mL syringe, draw the exact volume of solution to be injected, taking care to remove any air bubbles, as they may alter the administered volume.
  b. Remove the animal from its cage and immobilize it with the head oriented upward and aligned with the esophagus.
  c. Insert the gavage behind the incisors, directing it toward the posterior part of the throat.
  d. Gently advance the tube into the esophagus without force, allowing it to slide naturally without encountering resistance.
  e. Slowly inject the solution and carefully withdraw the tube.
  f. Return the animal to its cage.

***Note:*** In our experiment we use a dose of MET of 400 mg/kg (40 mg/mL, administered at 10 μL per gram of mouse body weight) every other day for 4 weeks.

**CRITICAL:** When drug administration is combined with IF, it must be administered under fasting conditions after 18 h from food removal.

11. Intermittent fasting (IF).

IF (complete withdrawal of food with free access to water) is defined as 24 h cycle of feeding and subsequent fasting from 6 p.m. to 6 p.m. of the following day (3 cycles a week) for 4 weeks.

12. At the specific time (we used 6 p.m.) remove all the food pellet from the cage.
13. After 24 h replace food pellet into the cage.

***Note:*** Ensure free access to water during fasting period.

**CRITICAL:** Water bottles may be dropped. Make sure the bottle is properly closed.

### Antitumor activity evaluation

**Timing: 5–10 min per mouse**

These steps include procedures to monitor tumor activity, both in terms of tumor growth and changes in animal body weight, which serve as an indirect assessment of treatment toxicity and the animals’ overall well-being. Moreover, the calculation of Treatment/Control (T/C%) and Increase of Life Span (ILS%) as recognized parameters to evaluate the antitumor activity of the drugs, and their efficacy intervals are defined.

14. Subcutaneous Tumor Measurement.
  a. Remove the animal presenting a subcutaneous tumor from its cage and restrain it in the operator’s hand to allow exposure of the tumor.
  b. Measure the length and width of the tumor by placing an open caliper on the tumor and gently closing it until it fits snugly around the tumor mass (without applying pressure).
  c. Read the measurements on the caliper.
  d. Calculate the tumor volume (TV) using the following formula:
    $$TV=\frac{d^{2}\times D}{2}$$
    where “d” is the width, and “D” is length. Both measurements are expressed in millimeters (mm) and have to be mutually perpendicular.
  e. Tumor volume can be converted to tumor weight assuming 1 mm^3^ = 1 mg.
15. Body weight measurement.
  a. Take an animal from the cage.
  b. Place the animal on the balance and record the weight once stable.
  c. Return the animal to its cage.

***Note:*** In our study, tumor volume and body weight were measured twice weekly, which was sufficient for accurate monitoring of the specific model used. The monitoring frequency should be adjusted on the experimental model.

16. Evaluate the antitumor activity of the subcutaneous models by calculating the T/C% (treated/control%), where T is the mean TV of the treated groups and C is that of the control group at each day of measurement. Considering the best T/C% evaluated during the activity, a T/C% value ≤ 40% is considered indicative of an active treatment.^2^^,^^4^
17. The Increase Life Span (ILS) is calculated as:

$$ILS\;\%=\frac{\text{ST}\;\text{treated}-\text{ST}\;\text{vehicle}}{\text{ST}\;\text{vehicle}}\times 100$$

ST: Survival time.

Treatment efficacy is interpreted as follows: ILS ≤ 40% = resistant tumor; 40% < ILS < 100% = responsive tumor; ILS ≥ 100% = highly responsive tumor. Animals are euthanized upon reaching ethical endpoints, in accordance with approved national regulations.

***Note:*** In our study, antitumor activity is evaluated both as a reduction in tumor volume (i.e., best T/C%) and as an increase in survival. Depending on the specific model, the operator may choose to use only one of these endpoints or other widely used parameters.

### Glycemia measurements

**Timing: 5–10 min per mouse**

These steps are performed on the day of randomization, and then during antitumor activity under treatment. The measurements of blood glucose are performed in each group of treatments and in vehicle-treated mice (at least 3 mice per group).

18. Blood glucose levels (mg/dL) are measured using clinical practice kits.
19. Insert the test strip provided by the kit into the designated automatic meter.
20. Disinfect the animal’s tail and obtain a drop of blood (approximately 10 μL) by puncturing the caudal vein with a needle (26G).
21. Apply the blood drop to the test strip.
22. After a few seconds, the meter calculates the blood glucose value, which can then be recorded.

### Collection of tumor and plasma samples from mice for downstream analysis

**Timing: 15 min per mouse**

This section outlines the procedures for the collection of snap frozen and FFPE tumors and plasma used for downstream assays.

23. Euthanize the donor animal by exposure to controlled increasing concentrations of CO_2_ (or other approved protocol).
24. Snap frozen and FFPE tumors collection.
  a. Disinfect the tumor area.
  b. Tumor excision.
    i. Make a small incision on the skin close to the tumor.
    ii. Carefully detach the skin from the tumor.
    iii. Excise the tumor and place it in a Petri dish containing saline solution.
  c. Tumor processing.
    i. Remove the external capsule of the tumor.
    ii. Discard necrotic or poorly cellularized portions.
    iii. Mince half of the tumor into homogeneous fragments of approximately 3 mm in diameter.
    iv. Snap freeze fragments in liquid nitrogen and fix one half of the tumor in 10% neutral buffered formalin for 24 h.
  d. Maintain snap frozen fragments at −80°C until use (preferably within 6–12 months) and embed the tumor formalin-fixed sample in paraffin or transfer into 70% ethanol until paraffin embedding (within one week).

**CRITICAL:** Perform these steps as quickly as possible to preserve all tumor properties.

25. Plasma collection.
  a. Open the animal’s thoracic cavity.
  b. Using a syringe, perform an intracardiac blood draw and collect the maximum possible volume.
  c. Transfer the blood into a citrate pre-filled tube and gently mix by slow inversion.
  d. Centrifuge at 4 °C at maximum speed (22000 *g*) for 5 min.
  e. Collect the plasma (the yellow fraction) and snap-freeze it for later use.

**Pause point:** After collecting all snap-frozen tumor and plasma samples, the subsequent steps can be scheduled at a later time. It’s recommended to performed all the downstream molecular analyses within 6–12 months.

### Metabolite extraction and sample preparation from tumor tissues

**Timing: 1–2 h.**

This section describes the extraction of metabolites from tumor samples under the detailed cold conditions to preserve metabolic integrity prior to FIA-HRMS analysis.

26. Weigh 20–50 mg of frozen tumor tissue from each animal, and record the exact weight of each specimen. Keep the sample in a frozen state throughout the procedure by keeping it on dry ice.
27. Homogenize the tissue using an Ultra Turrax (VWR, Pennsylvania, USA) by adding extraction solvent (85:15 MeOH/H_2_O) at a ratio of 10 μL per mg of tissue weight.
28. Store the homogenized sample at −80°C for 20 min.
29. Centrifuge at 4°C for 15 min at 13,000 *g*.
30. Collect the supernatants containing the metabolite extracts.
31. Store the supernatants at −80°C until metabolomics analysis.

***Note:*** Metabolites extract must be analyzed within two weeks.

### Untargeted metabolomics

**Timing: 2–3 min per run**

**Timing: 5–10 min**

This section describes untargeted Flow Injection Analysis-High Resolution Mass Spectrometry flow injection high resolution mass spectrometry (FIA-HRMS) analysis of metabolite extracts on a high-resolution Orbitrap Q Exactive mass spectrometer (Thermo Fisher Scientific) coupled to an HPLC system operated in flow-injection mode, enabling rapid acquisition in both positive and negative electrospray ionization.^5^ Metabolite features detected in full-scan mode are subsequently pre-processed and annotated using the EASY-FIA pipeline for high-throughput assignment of putative metabolite identities .^6^

32. FIA-HRMS approach.
  This step details FIA-HRMS acquisition of metabolite extracts using an HPLC system configured for flow-injection at low flow rate, without a chromatographic column, to maximize throughput while maintaining accurate mass measurements in both ionization polarities.
  The instrumentation for untargeted FIA-HRMS metabolomics consists of an Orbitrap Q Exactive mass spectrometer (Thermo Fisher Scientific) coupled to an HPLC system configured for flow-injection analysis. HPLC parameters should be optimized on the specific system used; final values can be reported in the Tables 1 and 2 below.
  a. Prepare 3 solvent mixtures for the HPLC (used in this protocol): A and B to make the isocratic flow, Needle Wash to rinse the needle used to inject the sample (Tables 3, 4, and 5). An identical set-up is available on all standard HPLC systems from major commercial vendors.
    ***Note:*** Solvent compositions are reported for a final volume of 1 L, which is sufficient for at least 200 FIA injections; prepare fresh mobile phases before each experiment by scaling to the required volume and adding ∼20% extra as safety margin.
  b. Set the HPLC system for flow-injection analysis with a constant flow rate of 50 μL/min and an injection volume of 8 μL per sample.
  c. Adjust the electrospray source parameters (e.g., source temperature, gas flows, nebulizer pressure) and document the final values in Tables 1 and 2 for positive and negative modes, respectively.
  d. Configure the mass spectrometer to acquire full-scan spectra from *m/z* 50–1,000 at a resolving power of 60,000 (at *m/z* 200) in both positive and negative ion modes, filling in the acquisition parameters in Tables 1 and 2 after method optimization.
  e. Enable continuous infusion of background ions as reference masses for internal calibration during the run (e.g., *m/z* 214.0896 in positive ionization mode and *m/z* 212.0750 in negative ionization mode) to maintain sub-ppm mass accuracy throughout the analytical batch.
  f. After each sample, inject two blanks consisting of isopropanol/water (60:40, v/v) for negative-ion runs or methanol/water (60:40, v/v) with 0.1% formic acid for positive-ion runs, to limit carry-over and reduce matrix-related ion suppression.
    ***Note:*** FIA-HRMS provides rapid profiling of a broad range of polar and semi-polar metabolites, but individual lipid species are typically interpreted at the class or summed-composition level because isobaric forms cannot be fully resolved without chromatographic separation.
33. FIA-MS/MS analysis.
  a. Prepare a pooled quality control (QC) sample by combining equal aliquots from representative extracts and use it for MS/MS acquisition.
  b. Perform HPLC analysis using LC and MS settings optimized for fragmentation experiments on the Orbitrap instrument (to be defined based on the specific system configuration), maintaining consistency with the full scan FIA conditions where possible.
  c. Acquire MS/MS fragmentation spectra in positive and/or negative ion mode using the same Orbitrap settings (e.g., resolving power, scan range) and stepped collision energies as defined for untargeted metabolomics MS/MS runs in the protocol, to support structural elucidation and library-based identification.
34. Data pre-processing with EASY-FIA.
  This step describes pre-processing of FIA-HRMS data using the EASY-FIA standalone application^2^ to perform blank subtraction, *m/z* feature alignment, and automatic first-pass annotation, yielding a single matrix of aligned metabolic features with their intensities suitable for statistical analysis.
  a. Export centroided mean mass spectra for each sample and its corresponding blank from the raw FIA-HRMS files using Xcalibur (Thermo Fisher Scientific) and save them as.csv files containing *m/z* and intensity values.
  b. Launch EASY-FIA and load the.csv files for all samples together with their matched blanks, ensuring that each sample has its corresponding blank in the acquisition list.
  c. In the EASY-FIA graphical user interface, set the *m/z* tolerance (Delta mass, ppm) according to the external calibration range of the mass spectrometer (QExactive set 6 ppm).
  d. Run the blank subtraction step: for each sample–blank pair, EASY-FIA searches for matching *m/z* values within the specified tolerance and subtracts the blank intensities from the corresponding sample intensities, optionally discarding features below the user-defined intensity threshold.
  e. Perform *m/z* alignment across samples using the ALIGNMENT function, which iteratively searches matching *m/z* values within the tolerance window, averages their masses, and constructs a single matrix of intensities with averaged *m/z* in the first column and sample-wise intensities in subsequent columns.
  f. If desired (positive ionization mode only), enable the “Sort matrix by adducts” option so that EASY-FIA groups and displays each putative [M+H]+ feature together with its corresponding [M+Na]+ and [M+K]+ adducts in sequential order, thereby facilitating downstream interpretation of adduct patterns.
  g. Apply the “Sample cut-off” option to remove features detected in fewer than a user-defined number of samples, and save the cleaned alignment matrix in both Excel and MATLAB formats (*suffix: _alignment_data or alignment_data_sorted, depending on whether the “Sort matrix by adducts” option is enabled*).
35. HMDB-based feature annotation with EASY-FIA.^6^
  This step provides first-round annotation of aligned *m/z* features by comparison with an in-house HMDB-derived database, generating an annotated intensity matrix for subsequent biological interpretation.
  a. In EASY-FIA, load the Excel file containing the aligned intensity matrix and select the appropriate in-house HMDB database file (HMDB_POS.mat or HMDB_NEG.mat) according to the ionization mode under investigation.
  b. Set the *m/z* tolerance for annotation and run the HMDB identification module, allowing EASY-FIA to search each experimental *m/z* value against the database and retrieve, when available, the corresponding HMDB ID, metabolite name, and adduct for each matching feature (6 ppm for Q Exactive instruments). If multiple matches are identified for a given feature, all possible annotations are reported.
  c. Save the annotated matrix as an Excel file (suffix _HMDB_ID), which contains the cleaned intensity matrix with associated metabolite identifiers and is used as input for subsequent multivariate and univariate statistical analyses.
  d. Perform final feature identification by integrating HMDB-based annotations with MS/MS fragmentation data obtained from the Q Exactive mass spectrometer (Thermo Fisher) and comparison with spectral libraries (ChemSpider, HMDB, and MIMEDB). Metabolite identities were manually curated and assigned MSI confidence levels (levels 1–4) according to the Metabolomics Standards Initiative guidelines.^7^

**Table 1:** MS source and acquisition parameters for untargeted metabolomics, positive mode

Source parameter	Value
Ionization mode	Positive
Spray voltage	3.8 kV
Sheath gas flow rate	25 L/min
Auxiliary gas flow rate	2 L/min
Sweep gas flow rate	1 L/min
Capillary temperature	320 °C
Aux gas heater temperature	100 °C

**MS acquisition parameter**	**Value**

MS1 resolution	35.000
Scan range	50–1000 *m/z*
AGC target (MS1)	3e^6^
Injection time (MS1)	50 *m/s*

**MS/MS acquisition parameter (Data-dependent mode)**	**Value**

MS/MS resolution	17.500
AGC target (MS/MS)	2e^5^
Injection time (MS/MS)	Automatic
Isolation window	1 *m/z*
Normalized collision energy (NCE)	16, 32, 48

**Table 2:** MS source and acquisition parameters for untargeted metabolomics, negative mode

Source parameter	Value
Ionization mode	Negative
Spray voltage	2.40 kV
Sheath gas flow rate	25 L/min
Auxiliary gas flow rate	3 L/min
Sweep gas flow rate	1 L/min
Capillary temperature	320 °C
Aux gas heater temperature	100 °C

**MS acquisition parameter**	**Value**

MS1 resolution	35.000
Scan range	50–1000 *m/z*
AGC target (MS1)	3e^6^
Injection time (MS1)	50 *m/s*

**MS/MS acquisition parameter (Data-dependent mode)**	**Value**

MS/MS resolution	17.500
AGC target (MS/MS)	2e^5^
Injection time (MS/MS)	Automatic
Isolation window	1 *m/z*
Normalized collision energy (NCE)	16, 32, 48

**Table 3:** Mobile phase A for LC-MS metabolomics, positive-mode

Reagent	Final concentration	Amount
Methanol	60.0%	600 mL
Water	40.0%	400 mL
Formic Acid	0.1%	11111 mL

**Table 4:** Mobile phase B for LC-MS metabolomics, negative-mode

Reagent	Final concentration	Amount
Isopropanol	60.0%	600 mL
Water	40.0%	400 mL
Ammonia	55555 mM	To pH 9

**Table 5:** Needle wash

Reagent	Final concentration	Amount
Acetonitrile	60.0%	600 mL
Water	40.0%	400 mL
Formic acid	0.1%	1 mL

### Targeted metabolomics of central metabolism

**Timing: 25–30 min per run**

**Timing: 90–180 min**

This section outlines the procedures for targeted metabolomic profiling of central metabolic pathways and describes the measurement of 83 metabolites, including amino acids and derivatives, nucleic acid–related compounds, vitamins, sugars, and intermediates of glycolysis and the tricarboxylic acid (TCA) cycle as reported in Table 10.

Metabolite levels were determined using a triple quadrupole mass spectrometer (LCMS-8060, Shimadzu) coupled to a UHPLC system. The mass spectrometer was equipped with an electrospray ionization (ESI) source operating in both positive and negative ion mode and in Multiple reaction monitoring (MRM) and polarity switching acquisition. Quantifier and qualifier transitions identified during method development are reported in Table 1. Chromatographic separation was performed using a Discovery HS F5-3 column (2.1 mm I.D. × 150 mm, 3 μm; Sigma-Aldrich) with a 20-min gradient, employing 10 mM ammonium formate at pH 3.5 as mobile phase A and acetonitrile as mobile phase B.

36. LC-MS analysis.
  a. Prepare two solvent mixtures for the HPLC, phase A and B, to make the analytical gradient (Tables 6 and 7).
    ***Note:*** Solvent compositions are reported for a final volume of 1 L; prepare fresh mobile phases before each experiment by scaling to the required volume and adding ∼20% extra as safety margin.
  b. Set a 20-min chromatographic gradient as follows (Table 8).
  c. Set the flow rate to 350 μL/min.
37. Mass spectrometry data acquisition.
  a. Acquire data in both positive and negative ion modes using Multiple reaction monitoring (MRM, and use the optimized MRM transitions for each metabolite, as reported in Table 10. The MRM transition, collision energies and declustering potential were automatically optimized using Shimadzu LabSolutions software.
  b. Perform analysis on a triple quadrupole mass spectrometer (LCMS-8060, Shimadzu) equipped with an electrospray ionization (ESI) source.
  c. Set source parameters as follows (Table 9): nebulizing gas flow rate 3.0 L/min; drying gas flow rate 15.0 L/min; desolvation line temperature 250 °C; heat block temperature 400 °C.
  d. Inject 1 μL of metabolite extract into a Discovery HS F5-3 column (2.1 mm I.D. × 150 mm, 3 μm; Sigma-Aldrich) at a column oven temperature of 40°C.

**Table 10:** Optimized MRM transitions for Targeted Metabolites

Name	Polarity	Quantifier transition	Qualifier transition	Retention time (min)	Collision energy (CE) (Quantifier transition/qualifier transition)	Declustering potential (DP) (Quantifier transition/ qualifier transition)
Cytidine 5′ monophosphate	+	324.00>112.05	324.00>95.00	1.4	−16.0/−46.0	−11.0/−11.0
Guanosine 5′-monophosphate disodium salt hydrate	+	364.00>152.05	364.00>135.00	1.8	−19.0/−41.0	−21.0/−22.0
Uracil	+	113.00>70.00	–	2.5	−14.0	−12.0
Adenosine 5′ monophosphate	+	348.00>136.05	348.00>97.10	3.0	−21.0/−51.0	−21.0/−22.0
Hypoxanthine	+	137.00>110.00	137.00>55.05	3.3	−22.0/−22.0	−18.0/−18.0
Uridine	+	245.00>113.05	–	3.5	−11.0/−36.0	−15.0/−16.0
Thymine	+	127.10>110.05	127.10>54.05	4.5	−22.0/−24.0	−18.0/−21.0
Inosine	+	269.10>137.05	269.10>118.95	4.6	−13.0/−36.0	−16.0/−16.0
Guanosine	+	284.00>152.00	284.00>135.00	4.6	−14.0/−32.0	−16.0/−17.0
Xanthosine dihydrate	+	284.90>153.05	284.90>135.95	4.56	−10.0/−27.0	−15.0/−16.0
Cytidine	+	244.10>112.05	244.10>95.00	1.2	13.0–16.0	22.0–20.0
Thymidine	+	243.10>127.10	–	4.6	−12.0	−14.0
Adenine	+	136.00>119.05	136.00>65.00	4.6	−24.0/−30.0	−17.0/−17.0
Adenosine	+	268.10>136.05	268.10>119.00	4.7	−17.0/−42.0	−16.0/−17.0
2′-Deoxycytidine	+	228.10>112.10	228.10>95.05	4.6	−13.0/−33.0	−14.0/−14.0
Uric Acid	-	167.10>123.95	167.10>96.2	2.3	17.0/20.0	11.0/29.0
Xanthine	-	151.00>108.00	151.00>42.00	3.4	20.0/ 35.0	16.0/18.0
Guanine	-	150.00>133.00	150.00>66.10	3.5	19.0/27.0	14.0 – 14.0
Niacinamide	+	123.10>80.05	123.10>53.10	4.7	−23.0/−23.0	−15.0/−16.0
D-(+)-Glucosamine hydrochloride	+	180.0>72.10	180.0>162.1	1.6	−15.0/−15.0	3.0/ 3.0
D-Gluconic acid sodium salt	-	195.2>74.9	195.2>129.05	1.2	20.0/ 15.0	22.0–20.0
L-Threonic acid hemicalcium salt	-	135.2>75.0	–	1.2	15.0/21.0	23.0/23.0
Sucrose	-	341.3>89.05	341.3>179.1	1.4	20.0/15.0	17.0/13.0
Nicotinic Acid	+	124.05>80.05	124.05>78.05	4.6	−23.0/−23.0	−15.0/−16.0
Choline chloride	+	104.10>60.05	104.10>45.10	3.0	−19.0/−29.0	−12.0/−12.0
D-Pantothenic Acid hemicalcium salt	+	220.10>90.15	220.10>72.05	4.6	−14.0/−11.0	−13.0/−14.0
Pyridoxal hydrochloride	+	167.90>150.05	167.90>94.15	4.6	−14.0/−24.0	−20.0/−10.0
4-Aminobenzoic Acid	+	138.25>65.10	138.25>77.10	5.7	−32.0	−14.0
Riboflavin	+	377.00>243.05	377.00>172.00	5.2	−23.0/−37.0	−12.0/−19.0
Biotin	+	245.10>226.95	245.10>97.05	5.2	−30.0/−26.0	−16.0/−16.0
Ergocalciferol	+	397.35>69.10	397.35>379.3	12.5	−29.0/−44.0	−15.0/−12.0
L-Ascorbic Acid	-	175.20>115.00	175.20>87.00	1.8	−11.0/−29.0	−18.0/−19.0
Cyanocobalamine	-	1354.50>1327.45	1354.50>1269.30	4.9	55.0/45.0	40.0/42.0
Pyridoxine	-	169.90>152.05	169.90>134.05	4.9	14.0	16.0
(±)-α-Lipoic Acid	-	205.20>171.10	205.20>127.10	9.8	8.0/14.0	14.0/22.0
DL-α-Tocopherol acetate	+	473.0>207.1	473.0>165.0	13.5	−21.0/−39.0	−17.0/−11.0
Cystine	+	241.00>73.90	241.00>151.95	1.1	−14.0/−15.0	3.0/3.0
L-Asparagine	+	133.10>87.15	133.10>28.05	1.1	−11.0/−29.0	−18.0/−19.0
L-Aspartic Acid	+	134.00>74.05	134.00>88.10	1.1	−15.0/−12.0	−19.0/−16.0
L-Serine	+	105.9>60.10	–	1.2	−13.0/−23.0	−11.0/−12.0
L-4-Hydroxyproline methyl ester hydrochloride	+	132.10>68.05	132.10>86.05	1.3	15.0/17.0	22.0/14.0
Glycine	+	75.90>30.15	–	1.2	−12.0/−45.0	−13.0/−10.0
D-Glutamine	+	145.20>127.15	145.20>190.00	1.1	−11.0/−22.0	−11.0/−25.0
L-Cysteine	+	122.00>76.05	122.00>56.05	1.3	−17.0/−26.0	−16.0/ −18.0
L-Threonine	+	120.10>74.15	120.10>56.05	1.3	−12.0/ −17.0	−15.0/−16.0
L-Methionine Sulfoxide	+	166.00>74.10	166.00>55.95	1.3	−13.0/ −23.0	−10.0/ −10.0
L-Glutamic Acid	+	147.90>84.10	147.90>56.10	1.2	−16.0/−25.0	−18.0/−10.0
D-Alanine	+	89.90>44.10	–	1.2	−14.0/−34.0	−11.0/ −11.0
L-Citrulline	+	176.10>70.05	176.10>159.05	1.3	−23.0/−12.0	−11.0/−11.0
L-Ornithine monohydrochloride	+	133.10>70.10	133.10>116.05	1.4	−17.0/−14.0	−18.0/−16.0
L-Proline	+	116.10>70.15	116.10>28.05	1.4	−16.0/−36.0	−17.0/−15.0
D-Lysine	+	147.20>130.10	147.20>56.10	1.1	−17.0/−14.0	−10.0/−10.0
L-Histidine monohydrochloride monohydrate	+	155.90>110.10	155.90>56.10	1.6	−15.0/−25.0	−18.0/−19.0
L-Arginine monohydrochloride	+	175.20>60.10	175.20>116.00	1.7	−23.0/−14.0	−12.0/−12.0
N-Acetyl-L-aspartic acid	+	175.90>134.05	175.90>88.05	1.3	−16.0/−22.0	−22.0/−10.0
4-Aminobutyric Acid	+	104.00>68.95	104.00>45.00	1.6	−14.0/−17.0	−12.0/−15.0
L- Glutathione reduced	+	308.00>179.10	–	1.4	−13.0/−17.0	−20.0/−17.0
L-Valine	+	118.00>57.10	118.00>55.10	1.6	−12.0/−23.0	−16.0/−16.0
D-Methionine	+	149.90>56.10	149.90>104.10	1.9	−16.0/−24.0	−17.0/−17.0
N-Acetylcysteine amide	+	164.10>122.10	164.10>59.05	2.5	−16.0/−22.0	−22.0/−10.0
L-Tyrosine	+	182.10>136.10	182.10>91.10	2.9	−26.0/−14.0	−11.0/−12.0
L-Isoleucine	+	132.10>69.15	132.10>86.20	2.2	−11.0/ −17.0	−19.0/−17.0
D-Leucine	+	132.10>30.05	132.10>86.05	2.5	−12.0/−18.0	−17.0/−19.0
D-Phenylalanine	+	166.10>103.10	166.10>120.10	4.6	−13.0/−27.0	−10.0/ −11.0
L-Kynurenine	+	209.10>192.05	209.10>94.10	4.7	−10.0/−19.0	−12.0/−13.0
Dl_Tryptophan	+	205.10>188.15	205.10>146.10	4.9	−10.0/−25.0	−13.0/−12.0
Cystathione	+	223.00>85.05	223.00>134.00	1.2	−27.0/−12.0	−13.0/−13.0
5-oxoproline	−	128.20>84.05	128.20>81.90	1.8	13.0/17.0	23.0/24.0
Hexose (D-(+)-Glucose)	−	179.20>89.10	179.20>59.05	1.3	9.0	21.0
Malate	−	133.10>114.95	133.10>71.15	1.4	16.0/16.0	12.0/13.0
Sodium Pyruvate	−	86.90>87.05	86.9>42.95	1.4	12.0	21.0
Sodium L-Lactate	−	89.30>89.05	–	1.6	14.0/13.0	15.0/14.0
Citrate	−	191.20>111.10	191.20>87.05	1.2	−23.0/−12.0	−11.0/−11.0
Succinic acid	−	117.30>73	117.30>99.05	1.9	13.0	22.0
Fumaric acid	−	115.0>71.10	115.0>26.95	4.6	12.0/13.0	13.0/27.0
4-Hydroxyphenyllactic Acid	−	181.20>163.00	181.50>135.10	4.9	15.0/17.0	22.0/14.0
Glyceric Acid (D-Glyceric acid calcium salt dihydrate)	−	105.20>75.10	105.20>57.05	1.2	13.0/14.0	17.0/19.0
2-Ketoisovaleric Acid	−	115.20>71.05	–	4.6	11.0	21.0
2-Isopropylmalic Acid	−	175.15>115.05	175.15>113.05	4.6	15.0/16.0	11.0/30.0
2-(2-Aminoethoxy)ethanol	+	62.15>44.10	62.15>27.10	1.7	−18.0	−30.0
Ethylenediamine	+	61.15>44.10	61.15>18.15	1.7	−11.0	−28.0
Putrescine	+	89.20>72.10	89.20>30.10	2.2	−13.0/−26.0	−12.0/−12.0
O-Phosphoethanolamine	+	142.10>44.20	–	1.2	−16.0	−19.0
Histamine	+	112.10>95.05	112.10>41.05	2.7	−17.0/−28.0	−15.0/−14.0

Adduct assignment: [M+H]+ for positive ionization mode and [M−H]- for negative ionization mode.
38. Data processing.
  a. Integrate chromatographic peak areas automatically using LabSolutions Insight LC–MS software (Shimadzu).
  b. Validate metabolite identity by matching retention times and quantifier-to-qualifier ion area ratios with those obtained from authentic reference standards.

***Note:*** The qualitative method could be converted into a quantitative approach through the use of isotopically labeled (deuterated) internal standards for each investigated compound, analyte-specific calibration curves, and appropriate analytical validation, including evaluation of linearity, precision, accuracy, matrix effects, and sensitivity.

**Table 6:** Mobile phase A

Reagent	Final concentration	Amount
Ammonium formate	10nM	0.63g
Water	∼100%	To 1L
Formic acid	To adjust to pH 3.5	As required

**Table 7:** Mobile phase B

Reagent	Final concentration	Amount
Acetonitrile	100%	1L

**Table 8:** LC parameters settings

Time (min)	%A	%B
0.0	100	0
1.40	100	0
3.50	75	25
7.50	65	35
10.30	5	95
13.70	5	95
13.80	100	0
20.00	100	0

**Table 9:** MS parameters settings

Parameters	Values
Nebulizing gas flow rate	3.0 L/min
Drying gas flow rate	15.0 L/min
Desolvation line temperature	250 °C
Heat block temperature	400 °C
Heating gas flow rate	10.0 L/min
Interface temperature	300 °C

### Targeted metabolomics of energy-related and redox-active cofactors

**Timing: 25 min per run**

**Timing: Data processing: 15–30 min (manual peak evaluation of 15 metabolites)**

This section outlines the procedures for targeted metabolomics by LC–MS/MS on metabolite extracts to quantify 15 metabolites involved in redox pathways and NAD metabolism (Table 15).

Metabolite levels were determined using a triple quadrupole mass spectrometer (LCMS-8060, Shimadzu) coupled to a UHPLC system. The mass spectrometer was equipped with an electrospray ionization (ESI) source operating in both positive and negative ion mode and in Multiple reaction monitoring (MRM) acquisition. Quantifier and qualifier transitions identified during method development are reported in Table 2. Chromatographic separation was performed using a X–Bridge BEH Amide column (2.1 mm I.D. × 150 mm, 3.5 μm; Waters) maintained with a 23-min gradient, employing 5 mM Ammonium bicarbonate at pH 9.5 as mobile phase A and acetonitrile as mobile phase B.

39. Chromatographic separation.
  a. Prepare 2 solvent mixtures for the HPLC (Tables 11 and 12): A and B make the analytical gradient.
    ***Note:*** Solvent compositions are reported for a final volume of 1 L; prepare fresh mobile phases before each experiment by scaling to the required volume and adding ∼20% extra as safety margin.
  b. Set a 23-min chromatographic gradient and apply the following gradient program (Table 13):
  c. Set a flow rate to 300 μL/min.
40. Mass spectrometry data acquisition (Table 14).
  a. Acquire data in both positive and negative ion modes using selected reaction monitoring (SRM), and use the optimized SRM transitions for each metabolite, as reported in Table 15.
  b. Perform analysis on a triple quadrupole mass spectrometer (LCMS–8060, Shimadzu) equipped with an electrospray ionization (ESI) source operating in both positive and negative ion modes. Set source parameters as detailed in Table 14.
  c. Inject 1 μL of each standard mix (1 μg/mL) into a X–Bridge BEH Amide column (2.1 mm I.D. × 150 mm, 3.5 μm; Waters) maintained at 40 °C oven temperature on a Shimadzu Nexera LC system. Maintain column re-equilibration under initial conditions prior to subsequent injections.
41. Data Processing.
  This section describes the integration of LC–MS/MS peaks, validation with pure standards, and the calculation of the relative energetic state.
  a. Integrate peak areas automatically with LabSolutions Insight LC–MS software (Shimadzu).
  b. Verify retention times and area ratios between quantifier and qualifier ions for all metabolites of interest using commercial pure standards (Sigma–Aldrich).
  c. Evaluate relative energetic states across experimental conditions using the Adenylate Energy Charge ratio,^8^ calculated from peak areas of ATP, ADP, and AMP as a relative indicator of cellular energy status (Figure 3B), according to the following equation:
    $$\text{Energy}\;\text{charge}=\frac{\left[\text{ATP}\right]+\frac{1}{2}\left[\text{ADP}\right]}{\left[\text{ATP}\right]+\left[\text{ADP}\right]+\left[\text{AMP}\right]}$$

**Table 11:** Mobile phase A

Reagent	Final concentration	Amount
Ammonium bicarbonate	5 mM	0.395g
Water	∼100%	To 1L
Ammonia	To adjust to pH 9.5	As required

**Table 12:** Mobile phase B

Reagent	Final concentration	Amount
Acetonitrile	100%	1L

**Table 13:** LC parameters settings

Time (min)	%A	%B
0	10	90
1.5	10	90
7	50	50
15.0	98	2
18	98	2
18.2 23	10 10	90 90

**Table 14:** MS parameters settings

Parameters	Values
Nebulizing gas flow rate	3.0 l/min
Drying gas flow rate	10.0 l/min
Desolvation line temperature	240 °c
Heat block temperature	400 °c
**Heating gas flow rate**	10.0 L/min
**Interface temperature**	300°c

**Table 15:** UHPLC- Mass spectrometry parameters for energy-related and redox-active cofactors

Name	Qualifier ion (*m/z*)	Quantifier ion (*m/z*)	Ionization	Retention time (min)	Collision energy (CE) (Quantifier transition/qualifier transition)	Declustering potential (DP) (Quantifier transition/qualifier transition)
Riboflavin	377>243	377>172.5	+	5.7	−22/−23	−21/−12
Flavin Adenine Dinucleotide (FAD)	785>348	785>136	+	5.6	−19/−36/−29	−40/−40/−40
Riboflavin 5′monophosphate (FMN)	456.8>439.2	456.8>359	+	5.7	−16/−22/−29	–
Niacinamide	123.05>80.2	123.05>78	+	1.9	−22/−23	−15/−15
Nicotinic Acid	124.2>78.15	124.2>80	+	1.8	−23/−22	−15/−14
Nicotinamide Mononucleotide	334.95>123.25	334.95>97.1	+	6.8	−15/−26	−19/−19
Guanosine 5′-monosphate (GMP)	361.95>79.05	361.95>211	-	6.9	23/18	13/13
Guanosine 5′-diphosphate (GDP)	441.9>344.15	441.9>159.05	-	7.1	20/28	17
Guanosine 5′-triphosphate sodium (GTP)	522.05>159.05	522.05>423.9	-	6.36	30/22	22
Adenosine-5′-monophosphate (AMP)	345.95>79	345.95>97.15	-	6.0	35/21	12
Adenosine 5′-diphosphate (ADP)	425.8>159.05	425.8>134.15	-	6.8	27/53/23	15/17/12
Adenosine 5′-triphosphate (ATP)	505.98>159.05	505.98>408.1	-	6.5	30/21/55	10/10/15
Nicotinamide adenine dinucleotide hydrate (NAD)	662.8>541	662.8>159.05	-	6.9	−41/−25/−18	−14/−16/−20
Nicotinamide adenine dinucleotide (NADH)	663.8>396.4	663.8>159.05	-	6.2	−17/−24	−24/−20
Nicotinamide adenine dinucleotide phosphate (NADP)	741.85>619.9	741.85>407.7	-	6.6	−46/−20/−27	−14/−22/−26

Adduct assignment: [M+H]+ for positive ionization mode and [M−H]- for negative ionization mode.

### DDP measurements in tumors and plasma samples

**Timing: 4 h**

This section outlines the procedures for measurements of platinum (Pt) into the tumors and plasma samples using an Inductively Coupled Plasma Mass Spectrometer (DRC-ICP-MS, NexIon 2000 B, PerkinElmer Instruments) equipped with a cyclonic spray chamber with a concentric nebulizer, a quadrupole mass filter and a dynamic reaction cell (DRC).

42. Pre-treatment tumor samples, before the analysis by DRC-ICP-MS.
  a. Weigh the samples with an analytical balance.
  b. Pre-treat the tumors in a microwave CEM model MARS-Xpress (CEM Corp., Matthews, NC, Table 16).
  c. Samples were digested by adding 1–5 mL of 65% HNO_3_ m/v (*Suprapur* Merck).
    After complete digestion and cooling, the digested solutions were transferred into graduated polypropylene tubes and diluted to a final volume of 10–20 mL with ultrapure water (*Milli-Q™* deionizing system, Millipore, Bedford, MA, USA).
  d. The diluted samples were subsequently analyzed by DRC-ICP-MS.
    ***Note:*** The microwave digestion system was used to reduce matrix interferences and minimize potential contamination during sample preparation. Microwave-assisted digestion also improved the speed and reproducibility of the analytical procedure. The system continuously monitored temperature at 1 s intervals using an infrared sensor and included a programmable exhaust system for the removal of corrosive fumes.
43. Pre-treatment plasma samples, before the analysis by DRC-ICP-MS.
  a. Transfer the plasma samples to graduated polypropylene tubes.
  b. Dilute with a 1% HNO_3_ solution (50- to 100- fold diluted solution for final analysis).
  c. After dilution, analyse samples by DRC-ICP-MS.
44. Analyse tumor and plasma samples by an Inductively Coupled Plasma Mass Spectrometer (DRC-ICP-MS), using the instrument operating parameters in Table 17.
  a. Optimize as in Table 17.
    ***Note:*** This instrument allows to obtain backgrounds values < 1 cps and detection limits of the order of ng/L (nanograms/L); moreover, the mass spectrometer allows to work in a linear way in a wide dynamic range, analyzing the analyte at different concentrations with comparable precision and accuracy.
  b. Quantitative analysis - Calibration curve.
    i. Perform calibration by “external calibration” method (6-point calibration curve).
    ii. Quantify Pt in Argon (Ar).
    iii. Prepare standard solution (calibration from 0.1 to 4.0 μg/L) from 10 mg/L Pt (ICP-MS calibration standard, Merck) by diluting with water (*Milli-Q™* deionizing system, Millipore) to produce similar concentrations of acids to those in the samples.
    iv. Curve fit: linear through zero.
  c. Quality control samples (QC).
    i. Quality control (QC) samples at low, medium, and high concentration levels (within the 0.1–4 μg/L Pt concentration range) are prepared independently from calibration standards by spiking the working standard solution with known amounts of analyte. At least two replicates were prepared for each QC level.
      ***Note:*** The results obtained confirmed the elevated linearity, sensitivity, precision and trueness of the method used and showed their suitability for the study of metals in the tumors and plasma samples.
  d. Calculate Pt concentrations.
    i. Calculate the concentration of Pt using the *Syngistix Software.* The software calculates the Pt concentration by comparing the element isotope intensities with the external calibration curve.
    ii. Calculate the final Pt concentration using the data obtained from the analysis of the sample, the reagent blank and the dilution factor used in the pre-analytical treatment.

**Table 16:** Operating parameters of the CEM microwave system (CEM)

Step	1	2	3	Cooling
Power (W)	100	150	200	–
Hold time (min)	2	2	1	10

**Table 17:** Instrument Operating Parameters for DRC-ICP-MS NexIon 2000B analysis

Analyte	Pt
Acquisition	Sweeps/readings 10, readings/replicates 3, number of replicates 3
Nebulizer	Meinhard
DRC Vented	Standard mode Cell q parameter 0.25 Cell a parameter 0
Dual detector mode	Pulse
Scan mode	Peak Hopping
MCA Channels	1
Dwell Time AMU	50 ms
Integration Time	500 ms
Torch Z position	0.00 mm
Standard - Nebulizer Gas Flow STD/KED [NEB]	0.96 L/min
Standard - Auxiliary Gas Flow	1.20 L/min
Standard - Plasma Gas Flow	15.00 L/min
Standard - QID Fixed Voltage	−13.00
Standard - ICP RF Power	1500.00
Standard - Analog Stage Voltage	−1675.00
Standard - Pulse Stage Voltage	1250.00
Standard - Q1 Rod Offset STD [QRO]	0.00
Standard - Cell Rod Offset STD [CRO]	−10.00
Standard - Cell Entrance/Exit Voltage STD	−2.00
Standard - Axial Field Voltage	0.00
Helium KED - KED Mode QRO	−12.00
Helium KED - KED Mode CRO	−15.50
Helium KED - KED Mode Cell Entrance Voltage	−3.00
Helium KED - KED Mode Cell Exit Voltage	−23.00
Helium KED - KED Mode Axial Field Voltage	475.00
Helium KED - Cell Gas B	7.00

### MET measurements in tumors and plasma samples

**Timing: 4 h**

This section describes the practical procedures for measuring MET concentrations in tumor and plasma samples using a UHPLC–MS/MS system consisting of an ExionLC™ Series UHPLC coupled with a SCIEX Triple Quad 3500 triple quadrupole mass spectrometer (AB SCIEX Italia s.r.l., Milan, Italy).

45. Plasma sample preparation procedure:
  a. Thaw the aliquots of standards and QC working solutions (WS) at 20°–25° C (approximately 20 min).
  b. Aliquot 50 μL of control plasma into two polypropylene 1.5 mL tubes to:
    i. prepare blank (i.e., matrix samples processed without addition of analyte or IS).
    ii. zero samples (i.e., matrix samples processed with addition of IS).
  c. Aliquot 45 μL of control plasma into a 1.5 mL polypropylene tube for calibration and QC preparation.
  d. Add 5 μL of each WS to the corresponding tube to obtain the following calibration curve points: 1, 5, 50, 100, 1000 and 5000 ng/mL; and QC levels: 10, 500 and 2500 ng/mL.
    ***Note:*** To prevent distortion of the matrix during the preparation of the calibration curve and maintain method integrity, ensure that the working solution (WS) (analyte in solvent) added to the control matrix remains below 10% of the total volume.
  e. Aliquot 50 μL of each study sample into individual tubes.
  f. Add 30 ng of ^2^H_6_-MET (as internal standard Internal Standard IS) to each tube and mix.
  g. Add 300 μL of cold Acetonitrile, then mix for 2 min to precipitate proteins.
  h. Centrifuge at 16.000 rcf for 10 min a 4°C to pellet the proteins.
  i. Transfer 200 μL of the supernatant into a clear LC micro-vial for UHPLC-MS/MS analysis.
46. Tumor sample preparation procedure:
  a. Remove tumor samples (200–300 mg) from the freezer and allow them to thaw for approximately 20 min.
  b. Weigh each tumor and homogenize it in phosphate-buffered saline (PBS) at a 1:3 (w/v) ratio using an Ultra-Turrax homogenizer (IKA, Staufen, Germany).
  c. Thaw aliquots of standards and QC working solutions at 20°–25° C for 20 min.
  d. Aliquot 150 μL of homogenized control tumor into two 2 mL polypropylene tubes for blank and zero samples.
  e. Aliquot 135 μL of homogenized control tumor into a 2 mL polypropylene tube for calibration and QC preparation.
  f. Add 15 μL of each WS to the corresponding tube to obtain the following calibration curve points: 4, 200, 400, 20′000, 80′000 and 200′000 ng/g; and Qc levels: 40, 500 and 10′000 ng/g.
  g. Aliquot 150 μL of each study tumor homogenate into individual 2 mL tubes.
  h. Add 60 ng of IS and mix.
  i. Add 900 μL of cold Acetonitrile and mix for 2 min for protein precipitation.
  j. Centrifuge at 13000 rpm for 10 min a 4°C.
  k. Transfer 200 μL of the supernatant into a clear micro-vial.
47. Set Chromatographic conditions.
  a. Injection volume: 5 μL.
  b. Autosampler temperature: 8°C.
  c. Analytical column: Kinetex 2.6 μm XB-C18 100 A˚ column, 100 × 2.1 mm, (Phenomenex, Torrance, CA, USA.
  d. Guard column: Kinetex XB-C18 VanGuard, 2.6 μm, 2.1 × 10 mm (Phenomenex, Torrance, CA, USA).
  e. Column oven Temperature: 37°C.
  f. MP-A: 6 mM ammonium formate, pH 4.5 ± 0.02, with 0.1% formic acid.
  g. MP-B: acetonitrile.
  h. Chromatographic separation was achieved using a linear–step gradient of solvent B at a constant flow rate of 0.40 mL/min, as follows:

**Table:** 

Time (min)	%A	%B
0.00	99	1
5.00	80	20
5.50	2	98
8.50	2	98
9.00	99	1
15.0	99	1
48. Mass spectrometry conditions.
  a. Mass spectrometer: 3500 Triple Quadrupole.
  b. Source: Turbo Ion Spray.
  c. Detection: MRM.
  d. Acquisition time: 15.0 min.
  e. Temperature: 550°C.
  f. Ionization mode: positive.
  g. Ion Spray voltage: 5.5 kV.
  h. Dwell: 79.0.
  i. Entrance Potential: 10.00.
  j. Collision Cell Exit Potential: 10.00.
  k. Curtain Gas: 35.00.
  l. Ion Source Gas 1 (Nebulizer Gas): 50.00.
  m. Ion Source Gas 2 (Heater Gas): 50.00.
  n. Collisionally Activated Dissociation (Gas): 9.00.
  o. Declustering Potential: 50.00.

**Table:** Compound parameter:

m/z	ID	Ce	
Metformin	130.0/71.0	quantifier	30
	130.0/60.0	qualifier	16
	130.0/85.0	qualifier	20
IS	136.0/77.0	quantifier	30
	136.0/60.0	qualifier	16
	136.0/85.0	qualifier	20

49. Quantitation condition.

Calibration Curve: linear fitting.

Weighting: 1/x^2^.

**Table:** 

Parameter	Metformin	IS
Transition Q1/Q3 (Da)	130.0/71.0	136.0/77.0
Smoothing Width	7	7
Retention time window (sec)	30	30
Expected retention time (min)	1.27	1.27

50. Data analysis.
  a. Use peak area ratios of calibration samples (MET peak area divided by IS peak area) to determine calibration curve parameters. Analytical data are acquired using Analyst 1.7.3.
  b. Calculate the intercept and slope of the calibration curve by weighted least squares regression.
  c. Assess calibration curve linearity by applying a weighted least squares approach using a 1/x^2^ weighting factor.
  d. Evaluate the goodness of fit of the linear regression model based on:
    i. The coefficient of determination (R^2^);
    ii. The back-calculated concentrations of individual calibration standards.
  e. Express the precision of QC samples as the coefficient of variation (CV%). The accuracy of calibration curve points and QCs is evaluated according to:
    $$\%Acc=\frac{x_{j}}{x_{i}}*100$$
    where:
    xj = found concentration of j-eme analyte.
    xi = actual concentration of j-eme analyte.
    ***Note:*** Acceptance criteria.^9^
  f. An analytical run is considered valid when:
    i. The accuracy of calibration standards is within ±15% of the nominal concentration (±20% for the lower calibration point). At least 75% of the calibration standards must meet this requirement.
    ii. Accuracy of QC samples is within ±15% for at least 67% of the QC samples and for at least 50% of the QC levels.

### Measurements of biochemical parameters in plasma samples

**Timing: 15 min/sample**

51. Thaw the snap frozen plasma sample in ice.
52. Load 100 μL of sample into the rotor and follow instruments instructions (Element RC, Scil). The “comprehensive plus” rotor allows the simultaneous measurements of the following parameters:

**Table:** Abbreviation

ALB	Abumin
ALP	Alkaline Phosphatase
ALT	Alanine Aminotransferase
AMY	Amylase
A/G	Albumin/globulin ratio
BUN	Blood Urea Nitrogen
BUN/CREA	BUN/creatinine ratio
CA	Calcium
CREA	Creatinine
GLOB	Globuline
K	Potassium
NA	Sodium
PHOS	Phosphorus
TBIL	Total Bilirubin
TP	Total Protein

53. Wait for the complete measurements.

### IHC on FFPE ovarian tumors

**Timing: 5 h**

Immunohistochemical staining is performed on 4 μm-thick sections of formalin-fixed, paraffin-embedded (FFPE) PDX ovarian cancer tumors. Use positively charged SuperFrost Plus slides (e.g., Epredia SuperFrost Plus Adhesion Slides, 10149870) for optimal tissue adhesion. The procedure is carried out using an automated immunostainer.

54. Deparaffinization and Heat Induced Epitope Retrieval (HIER).
  a. Fill heat-resistant containers (e.g., BioSB: Plastic Staining Dish, BSB7009) with Buffer H, pH 9, and cover them with aluminium foil.
  b. Prepare an equal number of containers (e.g., BioSB: Plastic Staining Dish, BSB7009) filled with distilled water (dH_2_O).
  c. Place all containers in a water bath with a support (e.g., BioSB: Staining Dish Support, BSB7086; TintoRetriever Digital Pressure Cooker, BS 7008) filled with dH_2_O and bring to boiling temperature (100°C).
  d. Once boiling is reached, immerse the slides in the containers containing Buffer H, pH 9, and perform HIER for 40 min at 100°C.
  e. At the end of incubation, transfer the slides to the containers containing dH_2_O, place them in a cold-water bath, and allow them to cool for 10 min to prevent thermal shock to the tissue sections.
55. Delimitation of tissue sections.
  a. Delineate the tissue sections using a PAP pen to limit reagent spreading during incubations.
56. Blocking of endogenous peroxidase activity.
  a. Cover the sections with 3% hydrogen peroxide (H_2_O_2_).
  b. Incubate for 10 min at RT.
  c. Wash the slides with PBS 1× containing 0.025% of Tween20.
57. Blocking of non-specific binding sites.
  a. Incubate sections with 10% normal animal serum diluted in PBS 1×.
  b. Incubate for 30 min at RT.

***Note:*** Do not wash after the blocking step. Remove excess serum by gently tapping or draining the slides.

58. Primary antibody incubation.
  a. Incubate sections with the primary antibody diluted in PBS containing 10% normal serum (according to the secondary antibody host). Antibody dilution is optimized for each specific marker:

**Table:** 

Antibodies	Dilution
Glutathione Peroxidase 4-GPX4	1:5000
4-hydroxynonenal-4-HNE	1:4000
Malondialdehyde-MDA	1:1500
anti-LAMP2	1:20000
p62	1:100
ACSL5	1:100
Ki67 sp6	1:500
Cleaved Caspase 3 9661	1:2000
  b. Incubate for 1 h and 30 min at RT.
  c. Wash slides with PBS 1× containing 0.025% of Tween20.
    ***Note:*** It is important to include positive controls for each primary antibody used, i.e. a tissue sample in which the presence of the target marker is confirmed.
59. Secondary antibody incubation.
  a. Incubate sections with biotinylated secondary antibodies diluted 1:200 in PBS 1×.
  b. Incubate for 30 min at RT.
  c. Wash slides with PBS 1× containing 0.025% of Tween20.
60. Antigen detection (ABC method).
  a. Incubate sections with VECTASTAIN Elite ABC-Peroxidase Kit (Vector Laboratories, cat. no. PK-6100), diluted 1:150 in PBS 1×.
  b. Incubate for 30 min at RT.
  c. Wash slides with PBS 1× containing 0.025% of Tween20.
61. Chromogen development.
  a. Prepare DAB (3,3-diaminobenzidine) solution (Vector Laboratories, cat. no. SK-4105) using 1 drop of DAB per 1 mL of diluent.
  b. Incubate sections for 5 min at RT.
  c. Stop the reaction by washing with distilled water (dH_2_O).
62. Nuclear counterstaining.
  a. Counterstain sections with Mayer’s Hematoxylin for 2 min.
  b. Rinse in running tap water for 5 min.
63. Dehydration and mounting.
  a. Ethanol 95%, 1 min.
  b. Ethanol 100%, 3 min.
  c. Ethanol 100%, 5 min.
  d. Xylene, 2 × 5 min.
  e. Mount coverslips using Micromount or an equivalent mounting medium.

## Expected outcomes

### Antitumor activity

The generation of tumor growth curves and body weight trends over time allows assessment of treatment efficacy on tumor growth and tolerance in vivo. In our study, we observed that the addition of IF enhanced the antitumor activity of the DDP + MET combination in an ovarian cancer PDX model with acquired resistance to DDP (Figures 1C and 1D), and significantly increased the overall survival (Figure 1D).

### Glycemia measurements

Under fasting conditions, a decrease in blood glucose levels is expected. Blood glucose is reported in mg/dL for each time point analyzed during the experiment. In our study, we observed a marked decrease in glucose levels during fasting, followed by recovery once food was reintroduced. Since we used nude mice without specific impairments in glucose metabolism (i.e., non-diabetic mice), the ability to compensate and regulate blood glucose was observed after the first cycle of IF, reflecting the mice’s metabolic capacity to buffer the rapid decrease in caloric intake.

### Measurements of biochemical parameters

**Figure 2:**
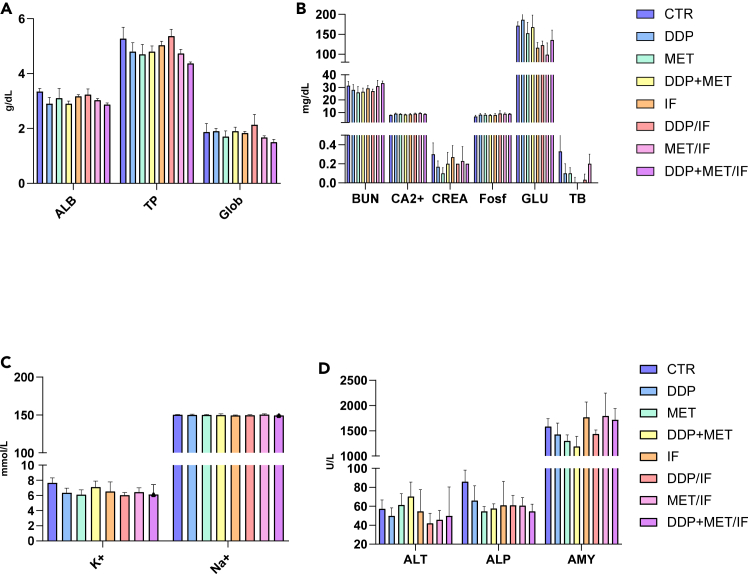
Blood and plasma measurements (A–D) Representative quantification of levels of different hepatic and kidney markers in the plasma of different treated and untreated mice. Values are mean±SD (n=3 biological replicates). ALB: albumin; TP: total protein; Glob: Globulin; BUN: Blood Urea Nitrogen; Ca2+: Calcium; CREA: Creatinin; PHOS: Phosphate; TB: Total Bilirubin; K+: Potassium; Na+: Sodium; ALT: Alanine Transaminase; ALP: Alkaline Phosphatase; AMY: Amylase test. Figure reprinted with the permission from Capellini et al, 2026.^1^

The measurements of biochemical parameters in plasma samples derived from the different treatment conditions would allow to have an indirect measurement of kidney and hepatic toxicity. The instruments directly get concentrations for each parameter (Figures 2A–2D).

### Immunohistochemistry

Immunohistochemical analysis is expected to reveal marker-specific staining with a clear cellular and subcellular localization, allowing comparison between control and treated PDX tumor samples. Treated samples (DDP+MET/IF) are anticipated to show quantitative and/or qualitative differences in staining intensity compared to controls, reflecting treatment-induced modulation of target protein expression.

### Mass spectrometry-based metabolomics

Mass spectrometry–based untargeted metabolomics is expected to provide comprehensive metabolic profiling of tumor samples, enabling the detection and relative determination of thousands of metabolites associated with the metabolic state of tumor tissue. This approach will facilitate the identification of metabolic signatures and pathways that are altered in cancer, potentially revealing novel biomarkers and mechanistic insights into tumor metabolism.

**Figure 3:**
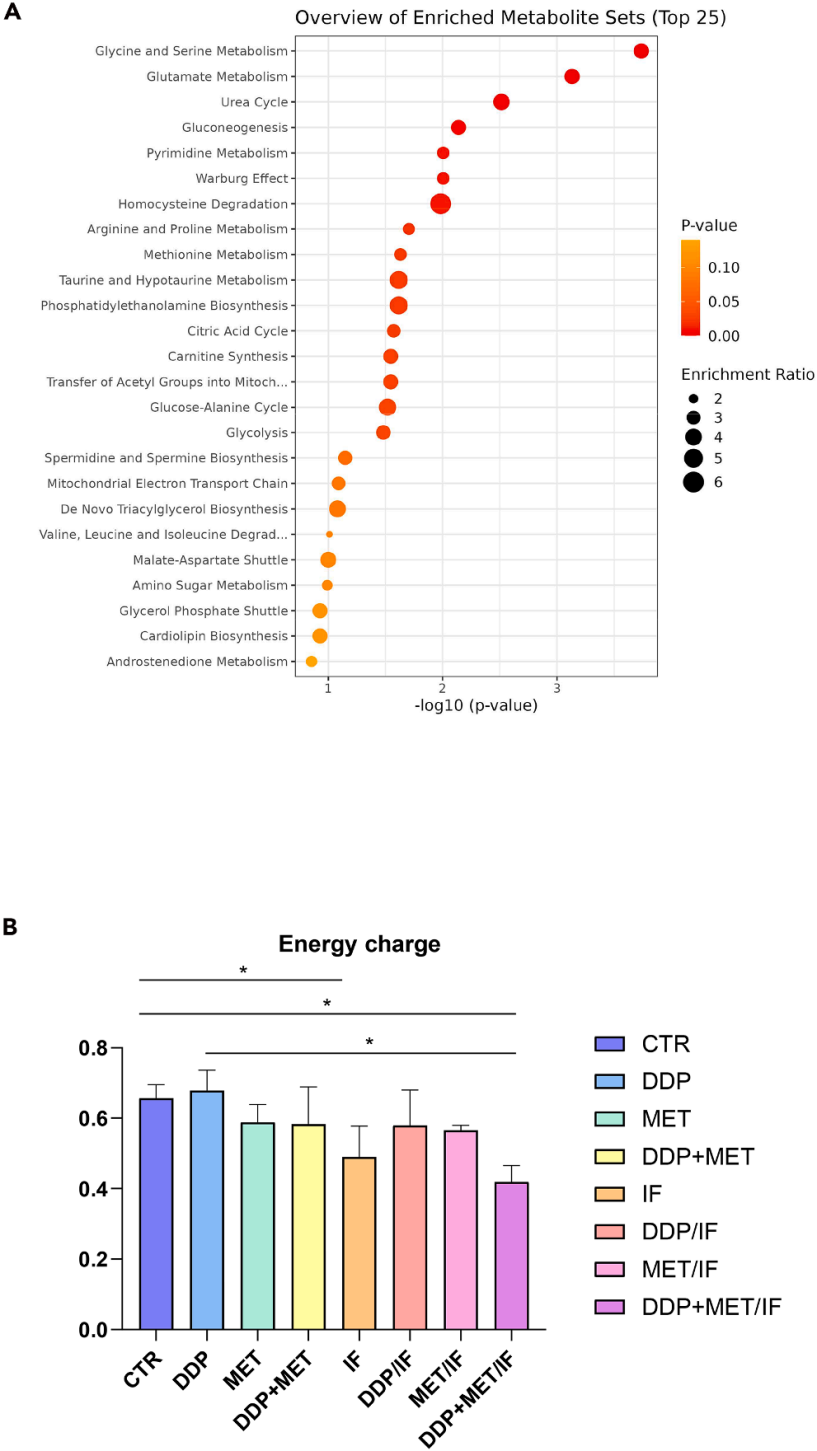
Metabolic pathway enrichment and tumor energy charge evaluations following DDP+MET treatment and intermittent fasting (A) Enriched metabolite set derived from statistically significant differential metabolites (CTR vs DDP+MET/IF) identified through both untargeted and targeted FIA-HRMS analyses. (B) Energy Charge of the tumors of mice in the different treatment groups fed with standard diet and IF. ∗ p < 0.05 (Kruskal-Wallis test). Figure reprinted with the permission from Capellini et al, 2026.^1^

Mass spectrometry–based targeted metabolomics is expected to enable the relative quantification of a predefined panel of metabolites selected to monitor specific metabolic pathways. Accurate measurements of these compounds will allow the calculation of metabolite ratios and pathway-specific indices, providing detailed information on the activity and regulation of the metabolic axes under investigation (Figure 3).

## Quantification and statistical analysis

Statistical analyses were performed by GraphPad Prism v6 software. The type of analysis as well as the number of biological replicates (n) for each group (represented as mean±SE) are reported in each figure legend. We used one-way ANOVA to evaluate differences among treatment groups and to detect overall differences between three or more independent groups. Tukey’s test (parametric) and Kruskal–Wallis test (non-parametric) were used for multiple comparisons. Animal survival was analyzed using Kaplan–Meier survival curves, and statistical significance was assessed using the Mantel–Cox test.

## Limitations

Regarding the use of these particular drug treatments and IF, this was specifically chosen for the use in an ovarian cancer PDX model with acquired resistance to platinum. The use of this treatment schedule can be limited to tumor’s models with a similar pharmacological background.

The FIA-HRMS approach enables rapid and high-throughput metabolic profiling of both polar and nonpolar compounds, providing a comprehensive overview of the metabolome. However, the lack of chromatographic separation represents an intrinsic limitation, as it reduces the ability to resolve isobaric and isomeric species and may increase matrix effects and ion suppression phenomena. In particular, lipids were considered at the class level rather than as individual molecular species due to the intrinsic limitation of the method in discriminating isobaric forms.

Regarding immunohistochemistry, the use of mouse monoclonal primary antibodies is generally not recommended on PDX tissues due to the risk of non-specific staining. Even in immunodeficient mice, endogenous mouse immunoglobulins can still be present in the tissue and may be recognized by anti-mouse detection systems. This can lead to background signal or false positives, as the secondary antibody binds both the primary antibody and endogenous IgG. To avoid this issue, primary antibodies raised in another species or specific mouse-on-mouse detection systems should be used.

Metformin quantification by UHPLC–MS/MS is feasible only within the validated calibration range, from the LLOQ to the ULOQ-specifically, 1–5000 ng/mL in plasma and 4–200000 ng/g in tumor tissue. These ranges may be extended by considering the accuracy acceptance limits (−20% at the LLOQ and +15% at the ULOQ), corresponding to approximately 0.8–5750 ng/mL in plasma and 3.2–230000 ng/g in tumor samples. Beyond these extended ranges, accurate quantification of the drug in the matrix cannot be ensured.

The analytical method for MET measurement developed in the specific PDX’s plasma and tumor is intrinsically restricted to these specific biological matrices. Because matrix composition can significantly influence extraction efficiency, ionization behavior, and overall analytical performance, applying the method to a different biological matrix cannot be assumed to yield comparable results. Therefore, before extending the method to alternative matrices, it is essential to evaluate potential matrix effects. This can be achieved by preparing calibration standards and quality-control samples in the new matrix and comparing their analytical responses with those obtained in the original matrix. While such effects must be experimentally assessed and validated, the use of a stable-isotope-labeled internal standard (e.g., deuterated or ^13^C-labeled) is expected to effectively compensate for matrix-related variability.

## Troubleshooting

### Problem 1

The addition of a fasting regimen may lead to significant body weight loss (“antitumor activity” paragraph, step 3).

### Potential solution

Monitor body weight frequently; ensure continuous water availability; consider pausing the IF cycle until body weight is recovered.

### Problem 2

While other animal procedures are quite standardized and easy to reproduce, blood collection from the animal via intracardiac puncture may be challenging and require experienced personnel (“collection of tumor and plasma samples from mice for downstream analysis” paragraph, step 3).

### Potential solution

To proper perform intracardiac blood collection perform cardiac puncture immediately after euthanasia. In addition, consider alternative blood-collection procedures. For instance, the retro-orbital blood collection from anesthetized animals in the protocol, as it may ensures a better yield. In general, operator-dependent procedures should be performed only by adequately trained personnel to minimize variability and animal distress.

### Problem 3

The excessive isoflurane exposure could cause a delayed in the post-anesthesia recovery and hypothermia (“tumor harvesting and Implantation” paragraph, step 5).

### Potential solution

The use of anesthesia must be carefully monitored by the operator, minimizing exposure time and anesthetic concentration as much as possible while ensuring sufficient time for tumor implantation. Animals should be continuously monitored for anesthesia depth and kept warm during recovery using infrared lamps or, when available, heating pads.

### Problem 4

Low reactivity due to improper fixation of the sample (“collection of tumor and plasma samples from mice for downstream analysis” paragraph, step 2).

### Potential solution

Ensure proper fixation by placing samples in 10% Neutral Buffered Formalin (NBF 10%) for no longer than 24 h. After fixation, transfer the samples to 70% ethanol until embedding. Samples should not exceed 10 mm in thickness to allow adequate penetration of the fixative. Use a leak-proof plastic container with a wide opening and maintain a sample-to-fixative volume ratio of at least 1:10.

### Problem 5

In the MET UHPLC–MS/MS quantification assay, analyte residue from a previous injection appears in subsequent chromatograms. This results in a signal increase that exceeds the acceptable carryover limit of 20% of the LLOQ peak area (“MET measurements in tumors and plasma samples” paragraph, step 2).

### Potential solution

Carryover in UHPLC–MS/MS is commonly caused by autosampler contamination, adsorption of sticky analytes, or retention within the flow path. Consider increasing and strengthening the needle and needle-seat wash cycles, extending the high-organic wash segment of the chromatographic gradient, and running blanks or null injections after high-concentration samples.

## Resource availability

### Lead contact

Further information and requests for resources and reagents should be directed to and will be fulfilled by the lead contact, Francesca Ricci (francesca.ricci@marionegri.it).

### Technical contact

Technical questions on executing this protocol should be directly to and will be answered by the technical contacts, Francesca Ricci (francesca.ricci@marionegri.it), and Laura Brunelli (laura.brunelli@marionegri.it).

### Materials availability

This study did not generate new unique reagents.

### Data and code availability

This study did not generate/analyze datasets or code.

## Acknowledgments

The research leading to these results has received funding from 10.13039/100020581AIRC under MFAG 2022 – ID. 27352 project – P.I. Ricci Francesca. The graphical abstract/figures were created using Biorender.com.

## Author contributions

Conceptualization, E.C., L.P., L.B., G.D., and F.R.; methodology, E.C., L.P., L.B., F.G., L.S., E.S., D. Cerullo, D. Corna, C.M., and F.R.; formal analysis, E.C., L.P., F.G., G.D., A.M.R., L.S., E.S., D. erullo, D .Corna, C.M., L.B., and F.R.; investigations, E.C., L.P., L.B., F.G., L.S., E.S., D. erullo, D. Corna, C.M., A.M., and F.R.; writing, E.C., L.P., L.B., F.G., G.D., and F.R.; funding acquisition, F.R.

## Declaration of interests

The authors declare no competing interests.
